# Inter-Reader and Inter-Modality Agreement for Radiographic and Magnetic Resonance Imaging Subchondral Bone Assessment in the Equine Proximal Phalanx Is Location- and Pattern-Dependent

**DOI:** 10.3390/ani16213493

**Published:** 2026-11-04

**Authors:** Maxime Vandersmissen, Alexandre Charles, Laurence Evrard, Claudia Fraschetto, Fabrice Audigié, Valeria Busoni

**Affiliations:** 1 Diagnostic Imaging Unit, Department of Equine Clinical Sciences, Faculty of Veterinary Medicine, University of Liège, 4000 Liège, Belgium; alexandre.charles@uliege.be (A.C.); levrard@uliege.be (L.E.); vbusoni@uliege.be (V.B.); 2 Clinique Vétérinaire de Grosbois, 94470 Boissy Saint Léger, France; vet.claudia.fraschetto@gmail.com; 3 ACAP3, Ecole Nationale Vétérinaire d’Alfort, 14430 Goustranville, France; fabrice.audigie@vet-alfort.fr

**Keywords:** horse, fetlock, proximal phalanx, subchondral bone, radiography, magnetic resonance imaging, sclerosis, resorption, stress fracture, inter-reader agreement

## Abstract

Standing low-field magnetic resonance imaging (MRI) is widely used for subchondral bone (SCB) assessment of the equine proximal phalanx, but radiography remains the most common first-line imaging modality, and no direct comparison between the two is available for SCB sclerosis and resorption. Fifty-four fetlocks from 52 horses referred for standing MRI were retrospectively assessed at the medial glenoid, sagittal groove and lateral glenoid, using an ordinal scale for sclerosis and a four-type nominal classification for resorption. Seven readers of varied clinical backgrounds performed independent radiographic readings; two board-certified radiologists then established a consensus reading, blinded to MRI. Agreement was quantified with Gwet’s agreement coefficients (inter-reader), weighted and unweighted Cohen’s κ (reader-MRI) and symmetric metrics (consensus-MRI). Inter-reader agreement for sclerosis was location-dependent (Gwet’s agreement coefficient = 0.27–0.73, lowest medially) and substantial-to-almost-perfect for resorption (Gwet’s agreement coefficient ≥ 0.72). The radiographic consensus aligned closely with MRI at the sagittal groove but underestimated marked sclerosis medially and over-called it laterally; fracture-type resorption was reliably detected at the sagittal groove but misclassified medially. Radiography can be relied upon for sagittal groove lesions, but complementary MRI is warranted medially when resorption or clinical suspicion of fracture is present, and to confirm radiographic positive calls laterally.

## Simple Summary

Bone injuries of the fetlock joint are a common cause of lameness in sport horses. Radiography is the primary imaging tool used to evaluate these injuries in general practice. Magnetic resonance imaging provides more detailed information but is only available on referral to specialised centres. Understanding how reliably radiographic findings reflect MRI findings is therefore relevant for clinical decision-making. In this study, seven veterinarians of different clinical backgrounds (general practitioners, sports medicine specialists and radiologists) interpreted radiographs of 54 fetlocks from 52 sport horses that had also been examined with standing magnetic resonance imaging. Agreement between readers and between the radiographic and magnetic resonance imaging interpretations was assessed at three anatomical locations of the proximal phalanx (medial, sagittal and lateral). Radiographic interpretation was less consistent between readers for some findings and locations, particularly for subtle bone density changes at the medial aspect. Overall, radiography remained informative but not equally reliable at every location. These results inform when radiography alone may be sufficient and when magnetic resonance imaging is warranted.

## 1. Introduction

Injuries to the proximal phalanx (P1) are a frequent cause of lameness and reduced athletic performance in horses, and they often involve the subchondral bone (SCB) plate and the adjacent trabecular bone of the proximal articular surface [1,2,3]. In sports and racing horses, SCB lesions develop due to chronic, repetitive overuse [2,4,5]. While SCB plate thickening can be considered adaptive to some extent [6], underlying trabecular bone sclerosis, bone oedema-like signal on magnetic resonance imaging (MRI) and bone resorption are typically observed in cases of bone failure, and may lead to stress fractures [2,7,8,9]. This adaptive-pathological distinction is particularly challenging at the medial glenoid cavity of the proximal phalanx, a preferential location of overload-related SCB thickening in sport horses [2,6].

MRI is used as the reference modality for assessment of subchondral and trabecular bone pathology in the equine fetlock due to its cross-sectional nature and its ability to detect bone oedema-like signal [4,10,11]. However, MRI requires specialised equipment and infrastructure, involves substantial cost, and therefore remains unavailable for many first-opinion practices. On the other hand, radiography is used as the first-line modality in ambulatory practice and equine hospitals, due to its ease of access, relative cost-effectiveness and excellent spatial resolution, the latter also offering greater sensitivity than MRI for certain imaging features such as periosteal reaction [2,6]. However, radiography is unable to detect bone marrow changes and abnormalities may only become apparent once structural alterations are advanced, limiting its ability to detect early or active lesions [12,13]. Furthermore, radiographic detection of SCB abnormalities depends on the conspicuity of the finding. Focal radiolucencies produce a locally conspicuous contrast change and are more readily identified when well-defined (cyst-like, fracture-type) than when amorphous, whereas increased opacity requires subjective comparison with the adjacent trabecular bone. Finally, because radiography is a two-dimensional projection, the exact angle of the dorsopalmar (plantar) view determines which portion of the curved proximal articular surface of the proximal phalanx is depicted tangentially: steeper descending angles project the more dorsal aspect of the SCB plate, while shallower angles depict a more palmar tangent. Small variations in projection angle therefore change which region of the SCB is best visualised on a given radiograph.

Computed tomography (CT) has been reported to be more sensitive than low-field standing MRI for the identification of some subchondral bone lesions in the equine fetlock [14] and would arguably provide a more directly comparable reference to radiography as both modalities derive their image from X-ray attenuation. However, standing CT is not yet as commonly available as standing MRI. The combination of radiography and standing MRI thus represents a frequent clinical setting in which fetlock pathology is investigated. In this context, understanding the diagnostic performance of the radiographic evaluation of P1 SCB, as well as its inter-reader reliability, is therefore clinically relevant, both for triage decisions (i.e., when to refer for MRI) and for determining how confidently positive radiographic findings can be considered.

The aims of the present study were to assess the diagnostic agreement of radiography and MRI for the identification of subchondral bone plate thickening and adjacent trabecular bone sclerosis (hereafter grouped as ‘SCB sclerosis’, as both findings reflect an adaptive densification of bone in response to biomechanical loading [5]) and subchondral bone resorption (hereafter ‘SCB resorption’) of proximal P1, and to evaluate whether reader-specific differences in radiographic interpretation exist.

Specific objectives were: (i) to quantify the inter-reader agreement of radiographic interpretation across readers of different clinical backgrounds; (ii) to quantify the agreement between individual readers and the MRI findings; and (iii) to assess inter-modality agreement between radiography and MRI.

We hypothesised that (i) inter-reader agreement for SCB resorption would be higher than for SCB sclerosis, with the lowest agreement expected for medial sclerosis; (ii) agreement between individual radiographic assessments and MRI would be higher among more experienced readers, but would remain low for medial sclerosis; (iii) inter-modality agreement would be location-dependent, but overall higher for resorption than for sclerosis, with radiographic detection of resorption being more reliable for cyst-like lesions and fractures than amorphous, poorly defined and/or small lesions.

## 2. Materials and Methods

### 2.1. Case Selection

Images of horses having undergone radiographic and MRI examinations of at least one fetlock at the Faculty of Veterinary Medicine of Liège between July 2022 and February 2026 were selected. Horses were included if a full MRI examination and at least one lateromedial and one dorsopalmar (plantar) radiographic projection were available.

### 2.2. Radiographs and MRI Acquisition

Radiographs were obtained using a direct digital detector (Agfa DR 10e C with Musica system, Agfa HealthCare NV, Mortsel, Belgium). At least one of the dorsopalmar (plantar) views was acquired with the cannon vertical and an approximately 20° descending angle [15] (variating slightly on a case-by-case basis depending on stance, fetlock positioning and individual horse conformation).

MRI images were obtained on the weight-bearing limb on standing, sedated horses, using a dedicated low-field (0.27T) open permanent magnet (Hallmarq Veterinary Imaging Ltd., Guildford, Surrey, UK), using at least the sequences of the standard MRI protocol of the hospital (Table 1).

Radiographs were acquired on the same day as the MRI examination, immediately prior to MRI acquisition.

### 2.3. MRI Reading

**Figure 1:**
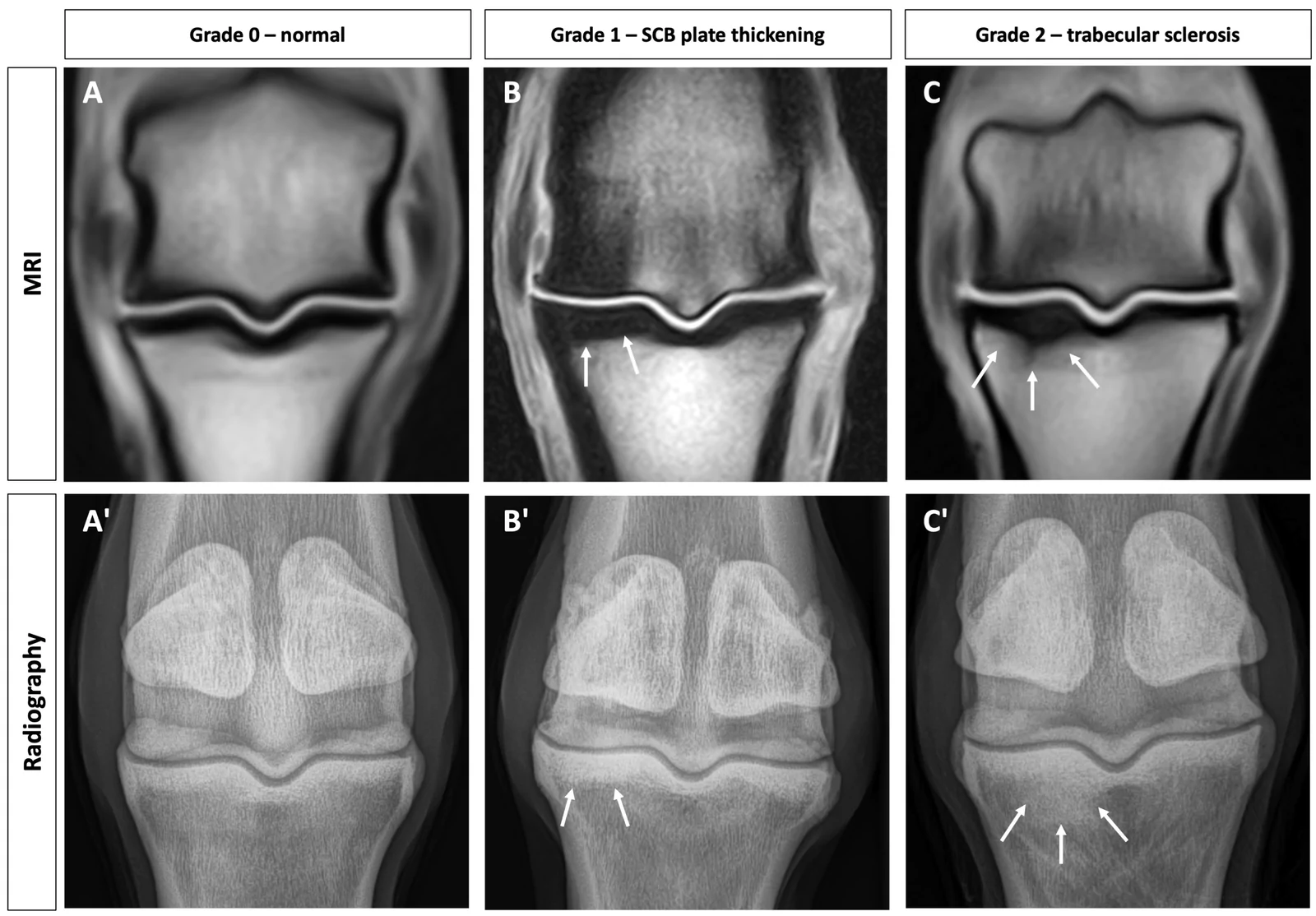
Grading scale for P1 SCB sclerosis—MRI and radiographic grades. Medial is to the left; MRI and corresponding radiographic image are from the same horse for each grade. The white arrows indicate the SCB sclerosis (**B**,**B’**,**C**,**C’**). Grade 0 = normal subchondral bone (SCB) plate (**A**,**A’**); Grade 1 = SCB plate thickening (**B**,**B’**); Grade 2 = SCB plate thickening with adjacent trabecular sclerosis (**C**,**C’**).

**Figure 2:**
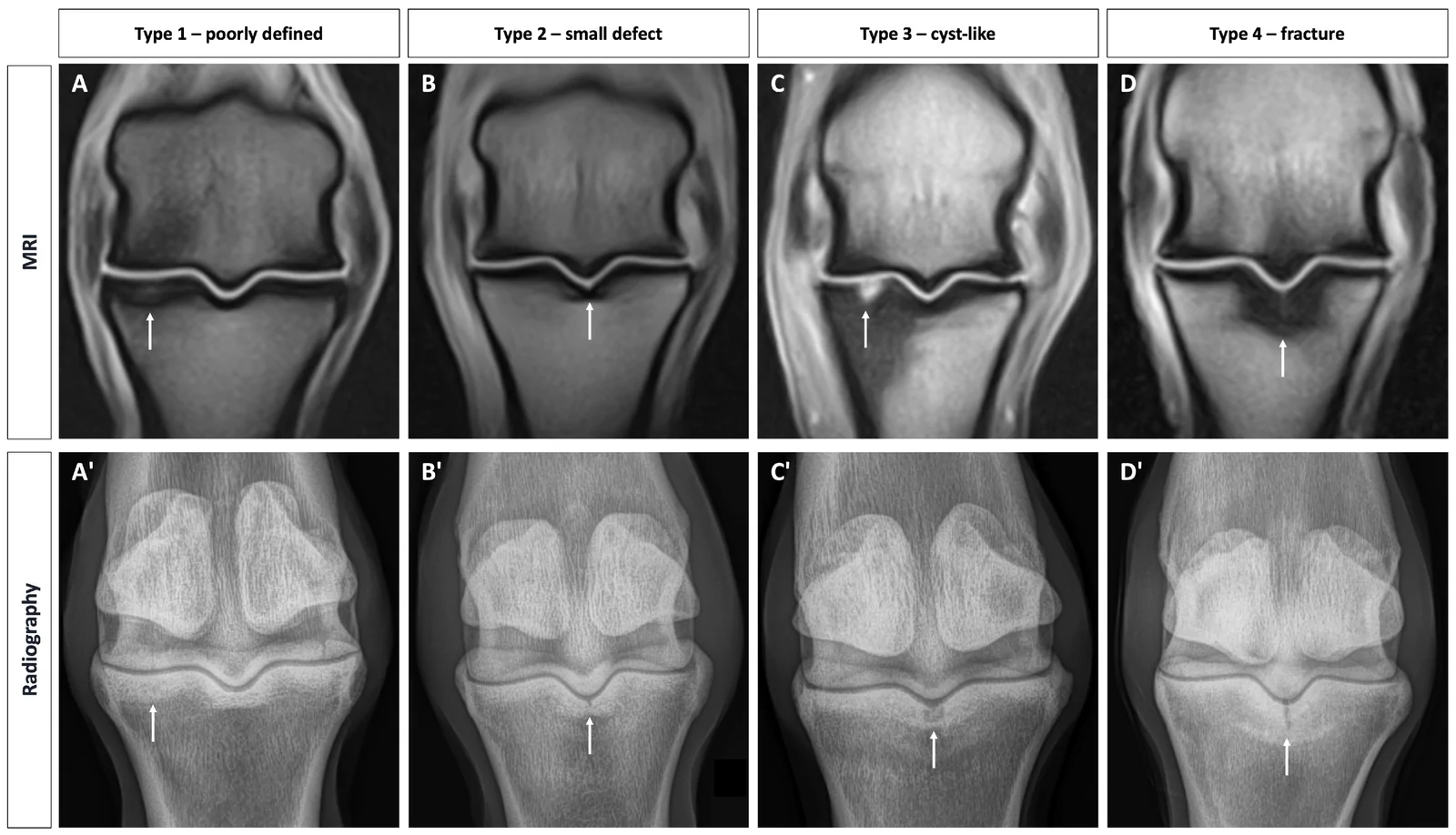
Pattern classification of P1 SCB resorption—MRI and radiographic types. Medial is to the left, MRI and corresponding radiographic image underneath are from the same horse, except for type 3 (no MRI cyst-like lesion was encountered in the study cohort; the MRI image shown for type 3 is provided for illustration only). The white arrow indicates the SCB resorption on each image. Type 1 = poorly defined ((**A**,**A’**)—at the medial glenoid); Type 2 = small ((**B**,**B’**)—at the sagittal groove); Type 3 = cyst-like ((**C**)—at the medial glenoid; (**C’**)—at the sagittal groove); Type 4 = fracture ((**D**,**D’**)—at the sagittal groove).

Three different P1 locations (medial glenoid, lateral glenoid and sagittal groove) were assessed in consensus on MRI images by 2 board-certified radiologists for sclerosis and resorption. Sclerosis severity was graded using a 3-point scale (Figure 1): grade 0—normal, grade 1—SCB plate thickening alone, grade 2—SCB plate thickening associated with adjacent trabecular sclerosis (reduced signal in all sequences). SCB resorption was classified into four distinct patterns (Figure 2): type 1—poorly defined (amorphous ill-defined hyperintensity in the SCB plate, type 2—small defect (small and shallow well-defined hyperintensity with disruption of the osteochondral interface, encompassing both focal defects and microfissures), type 3—cyst-like (well-defined round to ovoid hyperintensity of the SCB plate and/or adjacent trabecular bone), type 4—incomplete fracture (well-defined linear hyperintensity—type 4).

### 2.4. Radiographic Reading

A grading system for description of SCB sclerosis and a pattern-based classification of SCB resorption was established based on the MRI reading (Figure 1 and Figure 2). Sclerosis was graded as follows: grade 0—normal, well-defined SCB; grade 1—SCB plate thickening; grade 2—SCB plate thickening with loss of definition of the interface between the SCB plate and the trabecular bone and increased trabecular opacity. Resorption was classified as follows: type 1—poorly defined decreased opacity within the SCB plate; type 2—small well-defined disruption of the articular surface; type 3—cyst-like well-defined round to ovoid radiolucency of the SCB plate and/or adjacent trabecular bone, type 4—linear radiolucency/fracture extending from the articular surface into the SCB with or without extension into the adjacent trabecular bone.

Seven independent readers with different clinical backgrounds were recruited and grouped in three experience-based groups: two diplomates of the European College of Veterinary Sports Medicine and Rehabilitation—equine track (board-certified sports medicine specialists), two diplomates of the European College of Veterinary Diagnostic Imaging—large animal track (board-certified radiologists), and three equine general practitioners. Readers were provided with anonymized sets including a lateromedial and a dorsopalmar (plantar) view of every included fetlock.

A radiographic consensus was then built by a review of the two board-certified radiologists who discussed and reached a consensual grade, blinded to the MRI categorisation for every discordant location on radiographs.

### 2.5. Data Analysis

The choice of statistical methods followed recommendations of a recent review article on assessment of reader agreement in radiological studies [16]. All agreement and agreement coefficients were interpreted using the Landis–Koch scale [17]: values < 0.20 = poor/slight, 0.20–0.40 = fair, 0.40–0.60 = moderate, 0.60–0.80 = substantial, ≥0.80 = almost perfect.

Inter-reader radiographic variability was assessed with Gwet’s agreement coefficient: AC1 (unweighted) for the nominal resorption variable, and AC2 (linear-weighted) for the ordinal sclerosis grade.

Agreement between each individual radiographic reading and the MRI reading was assessed with linearly weighted Cohen’s κ (κw) for sclerosis and unweighted Cohen’s κ (κuw) for resorption. Svensson’s method was used to decompose sclerosis disagreement into systematic (relative position), scale-use (relative concentration), and reader-specific random (relative rank-variance) components. For resorption, Prevalence-Adjusted Bias-Adjusted Kappa (PABAK) complemented κuw to differentiate true poor agreement from low κuw values caused by uneven type prevalence.

Agreement between the radiographic consensus of the two board-certified radiologists and the MRI reading was quantified at each anatomical location using four symmetric metrics: the positive percent agreement (PPA; the proportion of MRI-positive cases concordantly identified by radiography), the negative percent agreement (NPA; the proportion of MRI-negative cases concordantly identified by radiography), and the forward and backward conditional agreements (FCA and BCA; analogues of the positive and negative predictive values). Because neither modality can be considered a true gold standard, these symmetric agreement metrics were preferred to the conventional sensitivity, specificity, positive predictive value and negative predictive value. Receiver-operating characteristic (ROC) curves and the corresponding area under the curve (AUC) were computed for the radiographic consensus grade as the ordering score, to characterise its discriminative ability against the MRI reading. Wilson-score 95% confidence intervals (CI) were computed for all proportions.

Inter-projection agreement was evaluated descriptively from each paired observation per reader, separately for sclerosis and resorption. For each reader and for the radiologist consensus, the percentage of identical paired readings was computed. Linearly weighted (sclerosis) and unweighted (resorption) Cohen’s κ values were determined for descriptive purposes only, given the limited per-reader sample. Aggregated transition tables across the seven readers were generated to visualise the magnitude and direction of any projection-related disagreement.

## 3. Results

### 3.1. Study Sample

Fifty-four fetlock examinations from 52 horses met the inclusion criteria. There were 27 mares, 21 geldings and 4 stallions, with a mean age of 9.5 years (ranging 1–27), predominantly Warmblood (BWP, SBS, Zangersheide, Selle Français, Oldenburg, KWPN and other Warmbloods—*n* = 43, Arabians—*n* = 3, Iberian breeds—*n* = 2, Thoroughbred—*n* = 1, unknown breed—*n* = 3). Lameness was localised to a forelimb in 42 horses and to a hindlimb in 10. Eight of the fifty-four fetlocks had two dorsopalmar radiographic projections available, yielding a total of 62 dorsopalmar projections read independently by each of the seven readers (434 reader-observations). When multiple dorsopalmar projections were available, only the standard 10–20° descending view [15] was retained for comparison with MRI, resulting in 54 radiographic observations per reader.

### 3.2. MRI Reading

Overall, 48/54 (89%) of the fetlocks presented at least one MRI abnormality across the three locations, while only 6/54 (11%) were free of both sclerosis and resorption.

On MRI, prevalence of SCB sclerosis was highest at the medial glenoid (63%, 34/54), followed by the sagittal groove (44.4%, 24/54) and the lateral glenoid (20.4%, 11/54). Trabecular sclerosis (grade 2) followed the same distribution: 29.6% (16/54) at the medial glenoid, 13.0% (7/54) at the sagittal groove, and 1.9% (1/54) at the lateral glenoid.

Prevalence of SCB resorption was highest at the sagittal groove (31.5%, 17/54), followed by the medial glenoid (16.7%, 9/54) and the lateral glenoid (1.9%, 1/54). Notably, every MRI-detected resorption case was associated with sclerosis at the same location, whereas sclerosis frequently occurred in isolation (25/34 medial glenoid, 7/24 sagittal groove, 10/11 lateral glenoid).

### 3.3. Radiographic Inter-Reader Agreement and Radiographic Consensus

#### 3.3.1. Sclerosis

For sclerosis, inter-reader agreement was best at the sagittal groove (substantial; Gwet’s AC2 = 0.73, 95% CI 0.63–0.83), intermediate at the lateral glenoid (moderate; AC2 = 0.56, 95% CI 0.45–0.66), and lowest at the medial glenoid (fair; AC2 = 0.27, 95% CI 0.17–0.37).

#### 3.3.2. Resorption

For resorption, inter-reader agreement was almost perfect at the lateral (Gwet’s AC1 = 0.96, 95% CI 0.92–0.99) and medial (AC1 = 0.85, 95% CI 0.78–0.91) glenoids, and substantial at the sagittal groove (AC1 = 0.72, 95% CI 0.62–0.82). Full multi-rater coefficients for both lesion types are also provided in Table S1.

#### 3.3.3. Radiographic Consensus

For the radiographic consensus, initial disagreement between the two board-certified radiologists was substantially more frequent for sclerosis than for resorption. Sclerosis discordances affected 29/54 fetlocks (54%) medially, 24/54 (44%) laterally, and 19/54 (35%) at the sagittal groove; resorption discordances affected 10/54 (19%) at the sagittal groove, 9/54 (17%) medially, and 0/54 laterally. All discordant cases were reviewed and reconciled through joint reading.

### 3.4. Agreement Between Individual and Consensus Radiographic Readings and MRI

Overall, agreement for sclerosis and resorption by individual reader and by reader group was location-dependent and revealed a modest effect of clinical background.

#### 3.4.1. Sclerosis

Per-reader and per-group agreement with MRI is detailed in Table S2. Per-reader Svensson decomposition values are shown in Figure S1 (source data in Table S3).

Individual agreement with MRI was location- and group-dependent. Board-certified radiologists achieved the highest mean κw at every location (0.44 medially, 0.40 laterally, 0.70 at the sagittal groove). Between-group differences were most pronounced at the medial and lateral glenoids: all reader groups showed only fair agreement at both locations, except for radiologists who reached moderate agreement at the medial glenoid. At the sagittal groove, all three groups reached similarly substantial agreement. Per-reader and per-group values are detailed in Table S2.

Svensson’s decomposition revealed distinct location-dependent patterns of disagreement. At the lateral glenoid, a systematic over-call of radiographic sclerosis relative to MRI was shared across readers (positive relative position). At the sagittal groove, the same directional over-call persisted but was moderate, with the largest disagreement component being individual rank-variance. At the medial glenoid, no consistent directional bias was observed, and disagreement was dominated by individual rank-variance. No observer disproportionately concentrated ratings on the extremes or the middle of the ordinal scale (near-zero relative concentration across locations). Per-reader Svensson decomposition values are shown in Figure S1 (source data in Table S3).

**Figure 3:**
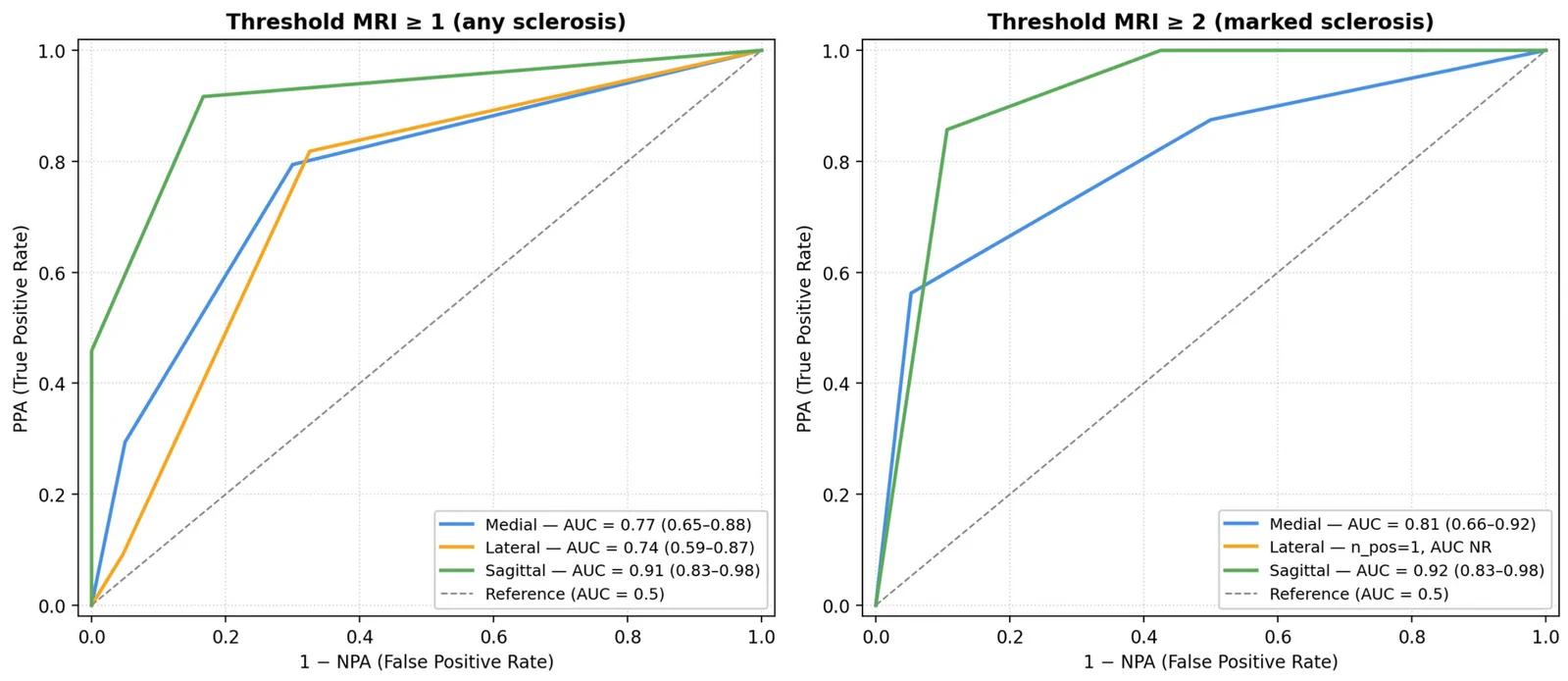
ROC curves for SCB sclerosis at the three anatomical locations, comparing the radiographic consensus grade against the MRI categorisation. **Left panel**: threshold MRI ≥ 1 (any sclerosis). **Right panel**: threshold MRI ≥ 2 (marked sclerosis). For each location, the AUC and its 95% confidence interval are indicated in the legend. The diagonal grey line represents the reference performance of a non-informative test (AUC = 0.50).

The radiographic consensus grading aligned well with the MRI categorization at all three locations (Figure 3). At the lateral glenoid, however, the AUC for marked sclerosis (MRI ≥ 2) could not be interpreted, as only a single MRI-positive case was available.

At the sagittal groove, agreement between the radiographic consensus and the MRI categorisation was almost perfect (PPA, NPA, FCA, and BCA all ≥ 81%). At the medial glenoid, agreement was substantial to almost perfect. At the lateral glenoid, agreement was low, with less than half of positive radiographic cases confirmed by MRI. When focusing on marked sclerosis (MRI ≥ 2), the radiographic consensus underestimated marked sclerosis at the medial glenoid (missing 44%, 7/16, of cases) and overestimated it at the sagittal groove; the lateral glenoid had too few cases (*n* = 1) to meaningfully estimate agreement. Complete agreement metrics by location and threshold are provided in Table S4.

#### 3.4.2. Resorption

For resorption, unweighted Cohen k values varied widely (−0.03 to 0.66) because of prevalence imbalance, while the prevalence-robust PABAK values were uniformly high across readers (substantial to almost perfect except for the sagittal groove for one reader). Between-group differences were minimal at the medial and lateral glenoids (similar PABAK values: medial 0.73–0.78; lateral 0.92–0.97). A group gradient was present only at the sagittal groove, which also showed the largest between-reader spread (0.79 vs. 0.51). Per-reader PABAK and κ values are detailed in Table S2. Per-reader discordant transitions with MRI are shown in Figure S2 (source data in Table S5).

For any visible resorption (MRI ≥ 1), the radiographic consensus showed an asymmetric agreement profile: high NPA but lower PPA at the medial glenoid and sagittal groove. In practice, the radiographic consensus reliably identified cases with no resorption but under-detected true positives, missing 44% (4/9) of MRI-positive cases at the medial glenoid and 24% (4/17) at the sagittal groove. Complete agreement metrics are provided in Table S6.

**Figure 4:**
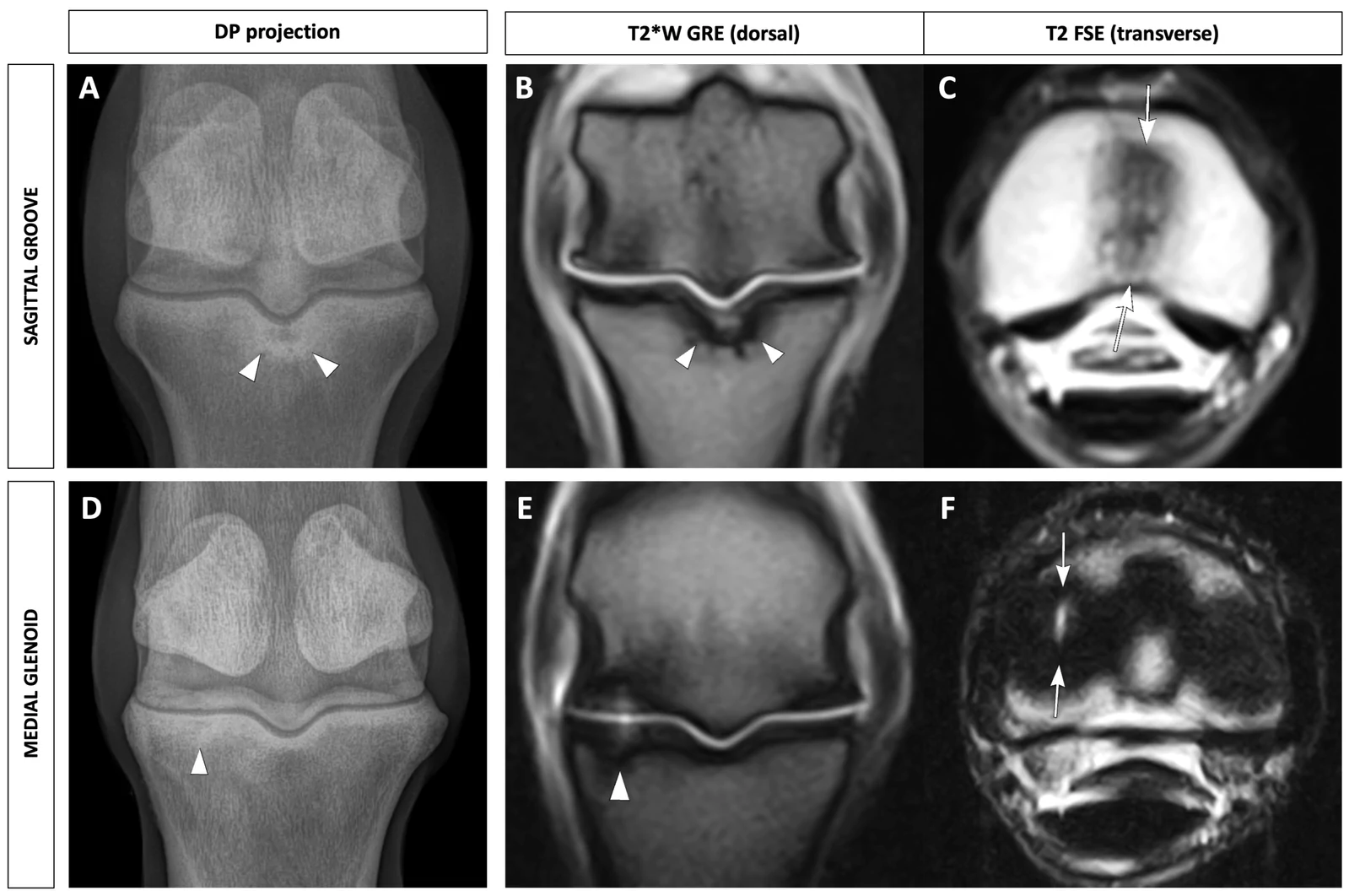
Comparison of radiographic and MRI appearance of subchondral bone resorption at the sagittal groove (top row) and medial glenoid (bottom row). Panels (**A**,**D**) show the dorsopalmar radiographs of two representative fetlocks; panels (**B**,**E**) show the corresponding T2*oW GRE dorsal MRI images; panels (**C**,**F**) show the T2W FSE transverse MRI images, which best demonstrate the dorsopalmar extent of the lesion, which was used as a parameter to classify a subchondral bone lesion in type 4 resorption. Top row (sagittal groove): a well-defined radiolucency interpreted as a cyst-like lesion by most readers on the radiograph ((**A**), arrowheads) corresponds on MRI to a linear, tripartite lesion (hypointense line bordered by 2 hyperintense lines) extending dorsopalmarly along the groove ((**B**), arrowheads; (**C**), arrows), consistent with a fracture rather than a true cyst-like lesion. Bottom row (medial glenoid): a region interpreted by most readers as a poorly defined demineralisation on the radiograph ((**D**), arrowhead) corresponds on MRI to a focal hyperintense fracture line ((**E**), arrowhead; (**F**), arrows), illustrating the radiographic under-detection of medial fractures in this cohort.

When analysed by resorption type, agreement between the radiographic consensus and the MRI categorisation was strongly type- and location-dependent. Discordances were largest for the most subtle patterns: poorly defined resorption (type 1) was often missed (PPA 33% medially, 0% at the sagittal groove) and small defects (type 2) were rarely correctly identified at the sagittal groove (PPA 29%). Fracture-type resorption (type 4) was reliably identified at the sagittal groove (PPA 88%, 7/8) but was systematically misclassified at the medial glenoid, where all three MRI-defined fractures were graded as poorly defined (type 1) on radiographs (PPA 0%, 0/3). At the sagittal groove, the single case classified as cyst-like (type 3) on radiographs corresponded to a type 4 (fracture) on MRI, and no MRI-defined type 3 was encountered. Per-pattern agreement metrics are provided in Table S7. Representative radiographic and MRI appearances of these resorption patterns are shown in Figure 4. A qualitative synthesis of the agreement findings across locations and lesion types is presented in Table 2.

### 3.5. Inter-Projection Agreement

Inter-projection agreement was assessed on eight fetlocks with two dorsopalmar projections, yielding 168 paired reader-observations (Figure S3).

#### 3.5.1. Sclerosis

For sclerosis, dichotomised as visible (grade ≥ 1) or not, discordance was highest at the medial glenoid (20/56 paired observations, 36%), intermediate at the sagittal groove (9/56, 16%), and lowest at the lateral glenoid (7/56, 13%). When analysed by grade, discordances almost exclusively involved grade 1 (plate thickening): grade 1 was seen on both projections in only 11 of the 54 pairs where it was reported, whereas grade 2 (trabecular sclerosis) was seen on both projections in 20 of the 37 pairs. The two-radiologist consensus reached 87.5% identical agreement, with a κw = 0.82.

#### 3.5.2. Resorption

**Figure 5:**
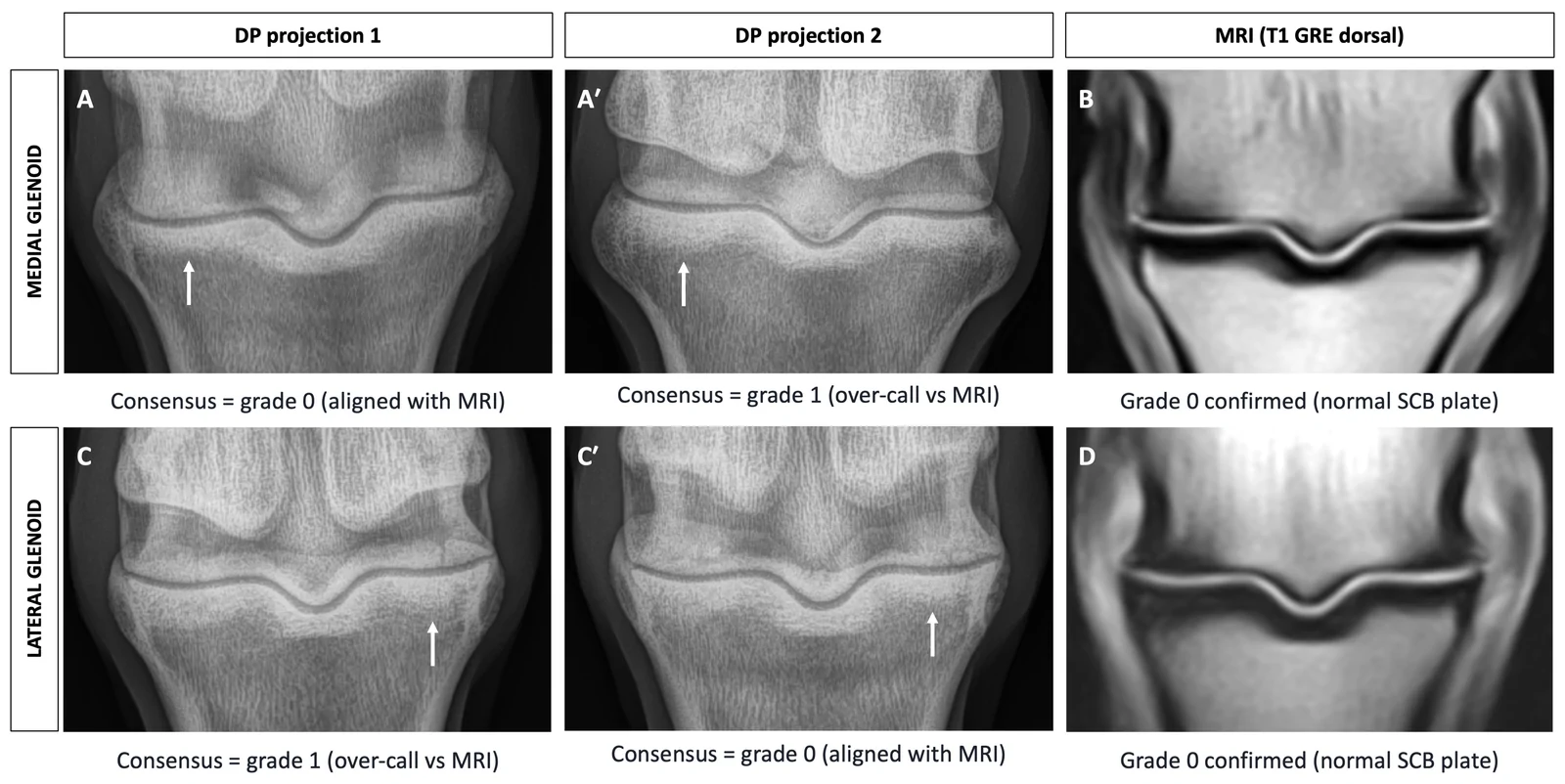
Angle-dependent radiographic interpretation of SCB sclerosis at the medial and lateral glenoids. Top row—at the medial glenoid: (**A**) dorsopalmar projection 1 aligned with MRI, the radiographic consensus graded 0 (arrow); (**A′**) dorsopalmar projection 2 of the same fetlock, the radiographic consensus graded 1 (arrow, over-call vs. MRI); (**B**) T1 GRE dorsal MRI confirms grade 0 (normal SCB plate). Bottom row—at the lateral glenoid: (**C**) dorsopalmar projection 1, the radiographic consensus graded 1 (arrow, over-call vs. MRI); (**C′**) dorsopalmar projection 2 of the same fetlock, the radiographic consensus graded 0 (arrow); (**D**) T1 GRE dorsal MRI confirms grade 0 at the lateral glenoid. Note that this fetlock has a genuine grade 2 sclerosis and type 1 resorption at the medial glenoid. Both fetlocks illustrate that radiographic detection of SCB sclerosis can be angle-dependent.

For resorption, dichotomised as visible (type ≥ 1) or not, discordance was concentrated at the medial glenoid (14/56 paired observations, 25%), with only 2/56 (4%) at the lateral glenoid and 0/56 at the sagittal groove (Figure 5). When analysed by type, discordances were driven by the most subtle patterns: poorly defined resorption (type 1) was seen on both projections in only 4 of the 20 pairs where it was reported. In contrast, fracture-type resorption (type 4) was identified on both projections in all 7 pairs where present. The two-radiologist consensus reached 87.5% identical agreement, with a κuw = 0.52.

## 4. Discussion

This study evaluates the agreement between radiography and MRI for the detection of SCB sclerosis and resorption at the proximal aspect of the P1 across readers with different levels of experience, as well as the agreement between a consensus radiographic assessment performed by 2 board-certified radiologists and MRI categorisation.

Inter-reader agreement for sclerosis was fair to substantial, lower than for resorption, and lowest at the medial glenoid. This likely reflects both the ordinal nature of the sclerosis scale and the poorly defined threshold between adaptive and pathological sclerosis, so individual readers effectively place “normalcy” at different levels. The medial glenoid, a preferential location for overload-related sclerosis [2,18], is particularly exposed to this ambiguity. In non-lame Warmblood showjumpers, subchondral bone thickening of the proximal phalanx is seen in nearly all limbs on both computed tomography and MRI, with asymmetric medial-over-lateral predominance, and was interpreted as adaptive modelling reflecting asymmetric physiological loading rather than pathology [19]. This directly parallels the distribution observed in the present study and suggests that some of the medial radiographic sclerosis may fall within this adaptive spectrum rather than reflecting pathology, in the absence of a clear threshold. This is also consistent with what is reported in high-level human athletes, where SCB sclerosis of the fingers in adolescent competition climbers has been explicitly regarded as a physiological adaptive stress reaction [20].

Individual agreement with MRI for sclerosis was location- and group-dependent, with board-certified radiologists achieving the highest mean κw at every location, supporting an experience/clinical background effect. However, within-group variability was comparable to the between-group gap, so experience alone poorly predicted individual performance. Svensson’s decomposition identified distinct location-specific patterns of disagreement, which the radiograph-MRI agreement results help explain.

At the lateral glenoid, a shared positive relative-position component reflected a systematic radiographic over-call, and agreement with MRI was correspondingly asymmetric, with over half of positive radiographic calls unconfirmed on MRI. This can be explained by the rarity of lateral lesions, which are less familiar to readers. An atypical or ambiguous appearance at an unfamiliar location may therefore be more readily called abnormal, producing a form of artefactual over-diagnosis rather than a true reader-dependent bias, consistent with its persistence even after consensus reading. A similar pattern of low radiographic specificity has been reported for SCB sclerosis assessment in canine elbows, where fair interobserver agreement and low specificity despite a moderate sensitivity has been attributed to overdiagnosis of sclerosis on radiographs [21].

At the medial glenoid, disagreement was instead dominated by reader-specific variability, and agreement with MRI was bidirectional, with both over-call of thickening and under-detection of trabecular sclerosis on radiographs relative to MRI. Consensus reading by the two radiologists recovered agreement, suggesting the disagreement stems from individual calibration on an ambiguous but familiar pattern, and can be reduced by targeted training (unlike the lateral over-call). This medial discordance is also consistent with the known superiority of MRI and CT over radiography for detecting SCB sclerosis [22,23]. Radiographic assessment of SCB thickening and sclerosis is inherently two-dimensional and angle-dependent, whereas MRI evaluates the SCB across its full extent in cross-section. This limitation of two-dimensional radiography is further supported by the lower inter-projection agreement observed for sclerosis (an angle-dependent effect that does not apply to MRI, and was not observed for resorption). The interpretive difficulty of SCB sclerosis is not confined to equine radiology: in a human study of hip osteoarthritis, interobserver agreement for SCB sclerosis grading was similarly limited on both radiographs and MRI, and MRI did not improve reader reliability [24]. Similar observations are reported in canine stifle osteoarthritis, where radiographic scores of SCB sclerosis correlated only moderately with MRI and were systematically higher on radiography [25]. The same mechanisms invoked by the authors [25] may also apply to the present study: the superimposition and geometric distortion inherent to 2D radiographic projections, and the possibility that low-field MRI itself under-detects subtle trabecular sclerosis that would still contribute to radiographic opacity.

At the sagittal groove, by contrast, minimal reader variability was observed, and agreement with MRI was almost perfect. Sagittal groove disease is a well-characterised entity with typical recognisable patterns [3,26], so opacity changes at this location are more readily detected. In addition, the sagittal groove suffers less superimposition than the glenoid cavities, as it extends deeper into the bone and is not overimposed with the thick palmar processes of the proximal phalanx.

Inter-reader agreement for resorption was substantial to almost perfect overall and higher than for sclerosis, given its nominal classification and its status as a more unambiguous, clinically relevant finding [4,5,9]. Agreement was almost perfect at the medial and lateral glenoids, but lower at the sagittal groove, probably reflecting greater morphological diversity at this location. The distinction between different morphologies can be difficult on two-dimensional radiographs, in particular between a type 2 (small defect) and a type 4 (incomplete fracture), which represent a lesion continuum [26].

For resorption, individual agreement with MRI showed no clear experience gradient, in contrast to sclerosis. Per-reader transition profiles showed distinct discordance patterns by location: at the medial glenoid, MRI poorly defined (type 1) resorption was frequently under-detected, consistent with radiography’s limited sensitivity to subtle opacity changes in thick SCB [12] and prior reports [2]. This pattern of under-detection of small or low-severity lesions is consistent with findings reported in non-lame sports horses, in which resorptive lesions of the proximal phalanx detected on CT (including the sagittal groove and the medial glenoid) were not identified on radiographs [19]. At the lateral glenoid, the low prevalence of lesions [1] precludes any firm conclusion, as only one MRI-positive case was available, with few discordances observed.

For radiographic consensus, agreement with MRI (any resorption, MRI ≥ 1) was asymmetric: a negative radiograph reliably ruled out MRI-positive resorption (high NPA), while some MRI-positive cases were missed radiographically (lower PPA), reflecting a tendency toward under-detection rather than over-call. Nonetheless, approximately a quarter of radiographically suspected resorptions were not confirmed on MRI, so even against this overall under-detection profile, a positive radiograph still warrants MRI confirmation, which is consistent with previously published work [4,13].

At the resorption pattern level, consensus-MRI agreement was strongly lesion-dependent, and the observed discordances are best understood in the context of comparing two-dimensional projections with cross-sectional imaging. At the sagittal groove, fractures were reliably identified; however, radiographic cyst-like findings always corresponded to underlying fractures rather than true cysts. This discrepancy likely represents an ambiguity in shape interpretation rather than a true diagnostic error: “cyst-like” is a descriptive, shape-based category, and on a two-dimensional dorsopalmar radiograph, a linear fracture line may not be visualised if it is not tangential to the x-ray beam. The superimposition of multiple confluent or overlapping fracture lines on radiographs may produce an ovoid radiolucent appearance rather than a linear one. At the medial glenoid, all three MRI-defined fractures were classified as poorly defined demineralisation on radiographs. Here again, the discrepancy is best attributed to the two-dimensional nature of radiography: a narrow fracture line running obliquely through the thick medial SCB plate may remain occult in the standard lateromedial and dorsopalmar views, particularly in the absence of a dedicated oblique projection [2]. In addition, medial glenoid fractures extended predominantly tangentially along the SCB plate, with only mild extension into the underlying trabecular bone. This configuration likely further contributes to the poorly defined radiographic appearance by producing broad superposition rather than a focal linear discontinuity. These findings could also suggest that poorly defined resorption and fracture may represent different stages of the same underlying process, similar to the sagittal groove [3], with practical clinical implications: a medial glenoid fracture may be radiographically occult because the available projections do not capture its plane, but also because poorly defined demineralisation itself may represent a prodromal sign of an incomplete fracture. Beyond the direct visualisation of the fracture line itself, the detection of periosteal reaction may have contributed to the better identification of fractures at the sagittal groove than at the medial glenoid. Periosteal reaction is a recognised prodromal sign of incomplete or stress fractures of the proximal phalanx [2,3,5,8,9]. When a sagittal groove fracture is present, periosteal reaction typically develops on the dorsal cortex [3] and is well visualised on the standard lateromedial projection; this feature was therefore available to the readers and may have supported the consistent identification of fracture-type resorption at this site, although it was not formally scored. When a medial glenoid lesion is present, by contrast, periosteal reaction tends to develop on the dorsomedial aspect of the cortex, which is not skylined on the standard dorsopalmar projection and may require a dedicated dorso~10°lateral-palmaromedial oblique view to be visible [2], which was not available to the readers. The bone marrow oedema-like signal detectable on MRI, which may accompany or precede structural subchondral bone changes, is itself a prodromal sign of fracture or active bone injury [2,3,5,8]. This signal was not recorded as a separate variable, as the aim was to compare lesion configuration between modalities. However, MRI readers cannot entirely disregard adjacent signal changes when categorising a lesion, which may have influenced how a given lesion was assigned to one morphological type rather than another. Therefore, the clinical context should also be considered when interpreting radiographic findings, as the likelihood of fracture may be weighted differently according to the severity and presentation of lameness. In a markedly lame horse, for example, a poorly defined resorptive lesion may raise greater suspicion for an underlying fracture, illustrating that MRI remains clinically necessary when a fracture or active bone injury is suspected.

For this study, we grouped into two configuration-based categories several MRI appearances of SCB resorption that have been described as separate entities in recent imaging classifications of equine sagittal-groove disease [3]. Type 2 (small defect) comprises lesions confined to the most superficial part of the SCB plate, including focal defects and microfissures. Published size thresholds for these entities are small, of the order of 1 to 3 mm in proximodistal extent [3], and typically fall within a single slice at the spatial resolution of standing low-field MRI (5 mm slice thickness, 0.6 mm inter-slice gap), where the linear versus punctate configuration of such lesions cannot always be reliably resolved. Type 4 (incomplete fracture) comprises continuous linear disruptions, extending either tangentially along the SCB plate across multiple slices, vertically into the underlying trabecular bone, or in both directions. Finally, as the primary aim of this study was to assess inter-modality agreement between radiography and MRI, a finer categorisation that may be meaningful on cross-sectional imaging cannot be matched by the two-dimensional nature of radiography; for this practical reason, these entities were pooled into broader, radiographically distinguishable categories.

Inter-projection agreement for resorption was high overall, with perfect agreement for the fracture pattern specifically. This likely reflects the greater dorsopalmar extent of sagittal groove fractures, which makes them detectable on more than one radiographic projection more easily, whereas small defects and more localised resorption are more likely to be missed if the single dorsopalmar view available is not tangential to the lesion. This may explain their comparatively lower detection rate on radiographs.

Several limitations of this study should be acknowledged. First, it reports agreement with an imaging comparator rather than accuracy against a true gold standard, as a retrospective study on clinical cases has no post-mortem confirmation of the lesions. The choice of low-field 0.27 T standing MRI as the comparator was clinically motivated but methodologically debatable, as MRI itself is likely to be observer-dependent in the fetlock region, similarly to what has been described in the foot [27], a bias limited here by MRI classification in consensus by 2 radiologists. Low-field MRI may also under-detect subtle trabecular sclerosis that would only be unequivocally assessed by quantitative CT or microCT [28,29]; some cases positive for sclerosis on radiographs but negative on MRI may therefore reflect genuine early trabecular sclerosis below the detection threshold of the low-field reference. A similar limit applies to resorption: MRI sensitivity for dorsoproximal osteochondral defects of the proximal phalanx has been reported at 52%, increasing with defect size [30], so some MRI-negative cases in this cohort may in fact host subtle resorption below the low-field detection threshold.

The radiographic set available for this study was limited to two orthogonal projections, which are routinely acquired prior to standing MRI at our institution, as part of the “MRI-package”, and correspond to a frequent screening situation. However, it should be acknowledged that a complete series including oblique projections is typically performed in clinical practice when fetlock pathology is suspected. The availability of these additional views might have modified radiographic detection and inter-modality agreement.

All radiographs were acquired by the same experienced technician using the same radiographic system, which minimised variability in image quality; however, agreement obtained under these conditions may not fully extrapolate to other settings (e.g., field radiography on portable equipment), where lower or more variable image quality could affect the detectability of sclerosis and resorption.

The cohort composition also limits several specific inferences: no MRI-defined cyst-like (type 3) resorption was encountered, so the overlap between radiographic cyst-like and MRI fracture classification could not be tested directly. Only one MRI-positive case of marked sclerosis (grade ≥ 2) was encountered at the lateral glenoid, rendering PPA and FCA estimates uninterpretable at that location. An additional limitation relates to the case selection: the cohort exclusively comprised fetlocks with clinical lameness referred for MRI, and only 6 of the 54 fetlocks were free of any sclerosis or resorption on MRI. This selection introduces a spectrum bias in two ways. First, a true normal population was not assessed, so the ability of readers to correctly identify normal SCB could not be adequately tested. Second, because each fetlock was likely to present at least one abnormality, the pre-test probability of finding medial glenoid SCB thickening (considered a non-specific change [19]) was inherently increased, which may partly explain the frequency of sclerosis calls at this location independently of true reader-based over-interpretation. Readers also interpreted the radiographs blinded to clinical information: while necessary to ensure objective inter-reader comparison, this design removes the clinical context usually available in routine practice, particularly relevant for the interpretation of medial SCB thickening and sclerosis, which may represent adaptive or pathological changes [6], or for the interpretation of poorly defined resorption. Additionally, readers participating in observer studies may perform differently and with greater inter-reader variability than in routine clinical practice, a well-documented “laboratory effect” [31]. In addition, the two board-certified radiologists were trained at and currently practice within the same institution; their shared reading habits may have introduced correlated biases that consensus reading cannot neutralise, with potential effect on the reference MRI categorization.

Finally, the inter-projection agreement analysis was based on only eight fetlocks with non-standardised projection angles and should be regarded as exploratory rather than definitive.

## 5. Conclusions

Radiographic evaluation of P1 subchondral bone sclerosis and resorption is diagnostically informative but shows location-dependent reliability compared with standing MRI. At the sagittal groove, both lesion types can be identified with confidence, with MRI mainly indicated to distinguish cyst-like lesions from fractures. At the lateral glenoid, a negative radiograph can be trusted, whereas positive findings warrant MRI confirmation because of a systematic radiographic over-call. At the medial glenoid, both over-call and under-detection are possible, and MRI should therefore be considered whenever clinical suspicion is high, in particular when a poorly defined radiolucency is seen, as this may represent an underlying incomplete fracture.

## Figures and Tables

**Table 1:** Standard MRI acquisition parameters for fetlock standing MRI. FSE = fast spin echo; GRE = gradient echo; INAV = intelligent navigator (motion compensation); NA = not applicable; STIR = short-tau inversion recovery; TE = echo time; TI = inversion time; TR = repetition time.

Sequence	TR (ms)	TE (ms)	Flip Angle (°)	TI (ms)	Number of Slices	Slice Thickness (mm)	Inter-Slice Gap (mm)	Scan Time
T1 GRE FAST	52	8	50	NA	8	5	0.6	1 min 51 s
T2* GRE FAST	68	13	28	NA	8	5	0.6	2 min 14 s
T2 FSE INAV	1500	80	90	15	8	5	0.6	2 min 42 s
STIR FSE INAV	2223	20	90	65–155	8	5	0.6	3 min 14 s–4 min 14 s

**Table 2:** Summary of radiographic agreement findings across the three anatomical locations of the proximal phalanx subchondral bone.

	Medial Glenoid	Sagittal Groove	Lateral Glenoid
**Sclerosis**			
Inter-reader agreement	Fair	Substantial	Moderate
Disagreement	Reader-specific	Low variability	Shared bias: systematic over-call
Effect of consensus	Substantially recovers agreement	Already high	Over-call persists
**Resorption**			
Inter-reader agreement	Almost perfect	Substantial	Almost perfect
Main discordance vs. MRI	Type 1 under detected	Types 1–2 under detected	Not applicable (low prevalence)
Fracture pattern	Read as poorly defined demineralisation	Reliably detected but sometimes read as cyst-like	Not applicable

## Data Availability

The data supporting the findings of this study are available from the corresponding author upon reasonable request.

## Supplementary Materials

The following supporting information can be downloaded at: https://www.mdpi.com/article/10.3390/ani16213493/s1, Figure S1: Svensson’s decomposition of reader-versus-MRI disagreement for subchondral bone sclerosis at the three anatomical sites (medial glenoid, lateral glenoid, sagittal groove); Figure S2: Discordant transition profile between individual radiographic readers and the MRI categorisation for subchondral bone resorption; Figure S3: Inter-projection agreement of radiographic grading across paired projection angles, for subchondral bone sclerosis (left panel) and resorption (right panel); Table S1: Multi-rater inter-reader agreement coefficients across the seven readers, by lesion type and anatomical site; Table S2: Per-reader and per-category agreement of radiographic readings with MRI, by lesion type and anatomical site; Table S3: Source data for Figure S1 (Svensson’s decomposition of sclerosis disagreement, per reader × site); Table S4: Inter-modality concordance between the radiographic consensus and the MRI categorisation for subchondral bone sclerosis, by anatomical site and severity threshold; Table S5: Source data for Figure S2 (Discordant transition profile for SCB resorption); Table S6: Inter-modality concordance between the radiographic consensus and the MRI categorisation for any SCB resorption (≥1) at each anatomical site; Table S7: Per-pattern concordance of the radiographic consensus with the MRI categorisation for subchondral bone resorption, by anatomical site and lesion pattern.

## Abbreviations

The following abbreviations are used in this manuscript:

AC1, AC2	Gwet’s agreement coefficient 1, 2
AUC	area under the curve
BCA	backward conditional agreement
CI	confidence interval
CT	computed tomography
FCA	forward conditional agreement
FSE	fast spin echo
GRE	gradient echo
MRI	magnetic resonance imaging
NPA	negative percent agreement
P1	proximal phalanx
PABAK	prevalence-adjusted bias-adjusted kappa
PPA	positive percent agreement
ROC	receiver operating characteristic
SCB	subchondral bone
