# Supplementary material for: Inter-Reader and Inter-Modality Agreement for Radiographic and Magnetic Resonance Imaging Subchondral Bone Assessment in the Equine Proximal Phalanx Is Location- and Pattern-Dependent
Source: Animals (Basel). 2026 Nov 4;16(21):3493. doi: 10.3390/ani16213493 (PMC13762259; doi:10.3390/ani16213493)
Supplement: Supplementary file 1 [file animals-16-03493-s001.zip › Figure_S1.pdf]

Figure 3 (N=54). Svensson's decomposition of sclerosis disagreement, per reader, per site

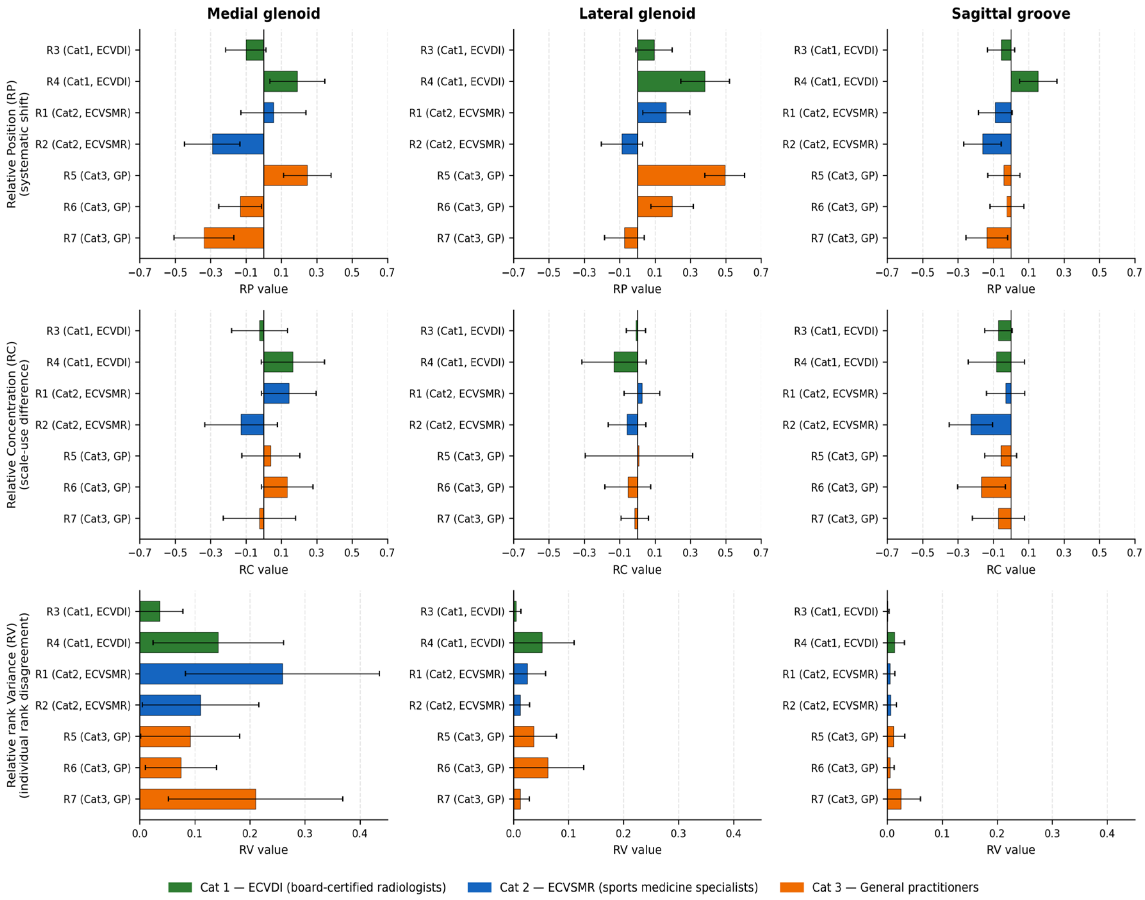

Figure S1. Svensson's decomposition of reader-versus-MRI disagreement for subchondral bone sclerosis at the three anatomical sites (medial glenoid, lateral glenoid, sagittal groove). Each panel shows the three components of disagreement per reader: RP (relative position, systematic directional shift between rater and reference), RC (relative concentration, differential use of the rating scale), and RV (relative rank-variance, residual non-systematic disagreement).
