# Supplementary material for: Inter-Reader and Inter-Modality Agreement for Radiographic and Magnetic Resonance Imaging Subchondral Bone Assessment in the Equine Proximal Phalanx Is Location- and Pattern-Dependent
Source: Animals (Basel). 2026 Nov 4;16(21):3493. doi: 10.3390/ani16213493 (PMC13762259; doi:10.3390/ani16213493)
Supplement: Supplementary file 1 [file animals-16-03493-s001.zip › Figure_S2.pdf]

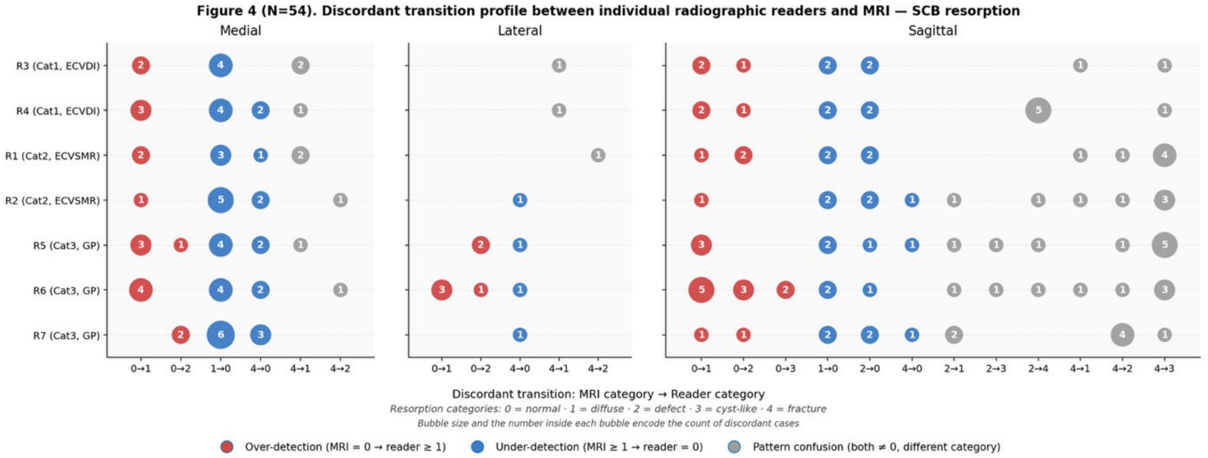


*Figure S2. Discordant transition profile between individual radiographic readers and the MRI categorisation for subchondral bone resorption. Each bubble represents the count of cases for which the MRI category was classified by the reader into a different category. Bubble size is proportional to the count of discordant cases, and the number inside each bubble corresponds to the count of discordant cases. Bubble colour indicates the direction of the discordance: red = over-detection; blue = under-detection; grey = pattern confusion.*
