# Supplementary material for: Inter-Reader and Inter-Modality Agreement for Radiographic and Magnetic Resonance Imaging Subchondral Bone Assessment in the Equine Proximal Phalanx Is Location- and Pattern-Dependent
Source: Animals (Basel). 2026 Nov 4;16(21):3493. doi: 10.3390/ani16213493 (PMC13762259; doi:10.3390/ani16213493)
Supplement: Supplementary file 1 [file animals-16-03493-s001.zip › Figure_S3.pdf]

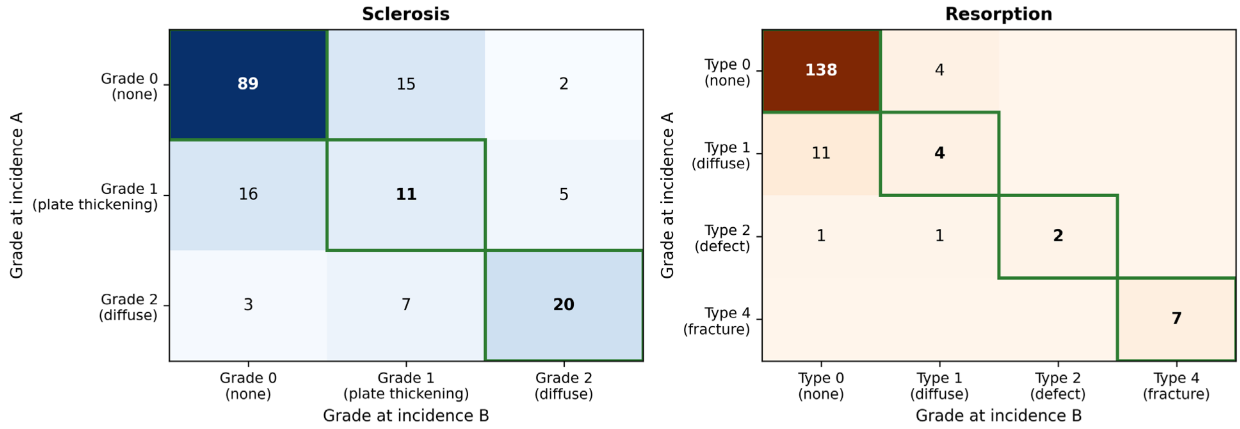


Figure S3. Inter-projection agreement of radiographic grading across paired projection angles, for subchondral bone sclerosis (left panel) and resorption (right panel). Each cell aggregates the 168 paired observations from the seven readers across the eight fetlocks and three anatomical sites. Rows correspond to the grade (sclerosis) or type (resorption) attributed at the first incidence; columns correspond to the second incidence. Diagonal cells (outlined in green) represent identical readings between the two incidences; off-diagonal cells represent disagreements. Colour intensity reflects the absolute number of paired observations in each cell.
