# Supplementary material for: Inter-Reader and Inter-Modality Agreement for Radiographic and Magnetic Resonance Imaging Subchondral Bone Assessment in the Equine Proximal Phalanx Is Location- and Pattern-Dependent
Source: Animals (Basel). 2026 Nov 4;16(21):3493. doi: 10.3390/ani16213493 (PMC13762259; doi:10.3390/ani16213493)
Supplement: Supplementary file 1 [file animals-16-03493-s001.zip › Table_S1.pdf]

Lesion type	Site	Coefficient	Value (95 % CI)
Sclerosis	Medial glenoid	Gwet's AC2 (linear weights)	**0.27 (0.17-0.37)**
Sclerosis	Lateral glenoid	Gwet's AC2 (linear weights)	**0.56 (0.45-0.66)**
Sclerosis	Sagittal groove	Gwet's AC2 (linear weights)	**0.73 (0.63-0.83)**
Resorption	Medial glenoid	Gwet's AC1 (unweighted)	**0.85 (0.78-0.91)**
Resorption	Lateral glenoid	Gwet's AC1 (unweighted)	**0.96 (0.92-0.99)**
Resorption	Sagittal groove	Gwet's AC1 (unweighted)	**0.72 (0.62-0.82)**

Table S1. Multi-rater inter-reader agreement coefficients across the seven readers, by lesion type and anatomical site. Cells are colour-coded according to the strength of agreement

**Poor / slight**	**Fair**	**Moderate**	**Substantial**	**Almost perfect**
< 0.20	*0.20 - 0.40*	*0.40 - 0.60*	*0.60 - 0.80*	*≥ 0.80*
