# Supplementary material for: Inter-Reader and Inter-Modality Agreement for Radiographic and Magnetic Resonance Imaging Subchondral Bone Assessment in the Equine Proximal Phalanx Is Location- and Pattern-Dependent
Source: Animals (Basel). 2026 Nov 4;16(21):3493. doi: 10.3390/ani16213493 (PMC13762259; doi:10.3390/ani16213493)
Supplement: Supplementary file 1 [file animals-16-03493-s001.zip › Table_S2.pdf]

Reader	Category	Sclerosis - κw			Resorption - PABAK		
		Med	Lat	Sag	Med	Lat	Sag
R3	Cat1	0.58	0.57	0.82	0.78	0.97	0.79
R4	Cat1	0.29	0.23	0.57	0.72	0.97	0.70
**Cat1 mean (n=2)**	—	**0.44**	**0.40**	**0.70**	**0.75**	**0.97**	**0.75**
R1	Cat2	0.21	0.31	0.68	0.78	0.97	0.70
R2	Cat2	0.24	0.20	0.57	0.78	0.96	0.70
**Cat2 mean (n=2)**	—	**0.23**	**0.26**	**0.62**	**0.78**	**0.97**	**0.70**
R5	Cat3	0.35	0.16	0.75	0.73	0.92	0.63
R6	Cat3	0.36	0.18	0.64	0.73	0.88	0.51
R7	Cat3	0.15	0.30	0.49	0.73	0.96	0.68
**Cat3 mean (n=3)**	—	**0.28**	**0.21**	**0.63**	**0.73**	**0.92**	**0.61**

Table S2. Per-reader and per-category agreement of radiographic readings with MRI, by lesion type and anatomical site. Sclerosis is summarised by the linearly weighted Cohen κ (κw); resorption by the prevalence-adjusted bias-adjusted κ (PABAK). Cat1 = two board-certified radiologists; Cat2 = two board-certified equine sports medicine specialists; Cat3 = three GP. Med = medial glenoid; Lat = lateral glenoid; Sag = sagittal groove. Cells are colour-coded according to the strength of agreement

**Poor / slight**	**Fair**	**Moderate**	**Substantial**	**Almost perfect**
< 0.20	*0.20 - 0.40*	*0.40 - 0.60*	*0.60 - 0.80*	*≥ 0.80*
