# Supplementary material for: Inter-Reader and Inter-Modality Agreement for Radiographic and Magnetic Resonance Imaging Subchondral Bone Assessment in the Equine Proximal Phalanx Is Location- and Pattern-Dependent
Source: Animals (Basel). 2026 Nov 4;16(21):3493. doi: 10.3390/ani16213493 (PMC13762259; doi:10.3390/ani16213493)
Supplement: Supplementary file 1 [file animals-16-03493-s001.zip › Table_S3.pdf]

Table S3. Source data for Figure S1 (Svensson's decomposition of sclerosis disagreement, per reader × site). RP = relative position (systematic directional shift; positive = over-call, negative = under-call); RC = relative concentration (scale-use difference); RV = relative rank-variance (individual rank disagreement); with 95% bootstrap confidence intervals. N = 54 fetlocks.

Reader	Category	Site	RP (95% CI)	RC (95% CI)	RV (95% CI)
**R3**	**Cat1 (ECVDI)**	**Medial**	-0.100 (-0.214, +0.014)	-0.023 (-0.181, +0.135)	+0.036 (-0.006, +0.078)
**R3**	**Cat1 (ECVDI)**	**Lateral**	+0.095 (-0.008, +0.197)	-0.008 (-0.062, +0.046)	+0.005 (-0.004, +0.014)
**R3**	**Cat1 (ECVDI)**	**Sagittal**	-0.056 (-0.133, +0.021)	-0.071 (-0.148, +0.007)	+0.001 (-0.002, +0.004)
**R4**	**Cat1 (ECVDI)**	**Medial**	+0.190 (+0.035, +0.345)	+0.165 (-0.014, +0.344)	+0.143 (+0.024, +0.261)
**R4**	**Cat1 (ECVDI)**	**Lateral**	+0.383 (+0.245, +0.521)	-0.132 (-0.314, +0.050)	+0.052 (-0.006, +0.110)
**R4**	**Cat1 (ECVDI)**	**Sagittal**	+0.154 (+0.049, +0.260)	-0.083 (-0.241, +0.076)	+0.014 (-0.004, +0.032)
**R1**	**Cat2 (ECVSMR)**	**Medial**	+0.055 (-0.129, +0.239)	+0.143 (-0.011, +0.298)	+0.259 (+0.083, +0.435)
**R1**	**Cat2 (ECVSMR)**	**Lateral**	+0.164 (+0.031, +0.296)	+0.026 (-0.076, +0.128)	+0.025 (-0.008, +0.058)
**R1**	**Cat2 (ECVSMR)**	**Sagittal**	-0.089 (-0.184, +0.007)	-0.030 (-0.138, +0.078)	+0.005 (-0.004, +0.014)
**R2**	**Cat2 (ECVSMR)**	**Medial**	-0.290 (-0.447, -0.133)	-0.128 (-0.332, +0.077)	+0.111 (+0.004, +0.216)
**R2**	**Cat2 (ECVSMR)**	**Lateral**	-0.087 (-0.203, +0.029)	-0.058 (-0.165, +0.049)	+0.013 (-0.004, +0.029)
**R2**	**Cat2 (ECVSMR)**	**Sagittal**	-0.161 (-0.266, -0.056)	-0.228 (-0.350, -0.105)	+0.006 (-0.004, +0.017)
**R5**	**Cat3 (GP)**	**Medial**	+0.247 (+0.113, +0.381)	+0.041 (-0.123, +0.204)	+0.092 (+0.002, +0.182)
**R5**	**Cat3 (GP)**	**Lateral**	+0.495 (+0.383, +0.608)	+0.009 (-0.295, +0.313)	+0.037 (-0.004, +0.078)
**R5**	**Cat3 (GP)**	**Sagittal**	-0.040 (-0.131, +0.051)	-0.058 (-0.148, +0.033)	+0.012 (-0.009, +0.032)
**R6**	**Cat3 (GP)**	**Medial**	-0.132 (-0.253, -0.012)	+0.133 (-0.013, +0.279)	+0.075 (+0.010, +0.139)
**R6**	**Cat3 (GP)**	**Lateral**	+0.197 (+0.076, +0.317)	-0.054 (-0.182, +0.074)	+0.063 (-0.003, +0.128)
**R6**	**Cat3 (GP)**	**Sagittal**	-0.023 (-0.119, +0.072)	-0.167 (-0.302, -0.032)	+0.005 (-0.002, +0.013)
**R7**	**Cat3 (GP)**	**Medial**	-0.338 (-0.507, -0.170)	-0.023 (-0.228, +0.181)	+0.210 (+0.052, +0.369)
**R7**	**Cat3 (GP)**	**Lateral**	-0.073 (-0.186, +0.040)	-0.014 (-0.092, +0.064)	+0.012 (-0.004, +0.029)
**R7**	**Cat3 (GP)**	**Sagittal**	-0.137 (-0.255, -0.019)	-0.071 (-0.217, +0.076)	+0.025 (-0.011, +0.061)
