# Supplementary material for: Inter-Reader and Inter-Modality Agreement for Radiographic and Magnetic Resonance Imaging Subchondral Bone Assessment in the Equine Proximal Phalanx Is Location- and Pattern-Dependent
Source: Animals (Basel). 2026 Nov 4;16(21):3493. doi: 10.3390/ani16213493 (PMC13762259; doi:10.3390/ani16213493)
Supplement: Supplementary file 1 [file animals-16-03493-s001.zip › Table_S4.pdf]

Site	Threshold	PPA % (95% CI)	NPA % (95% CI)	FCC % (95% CI)	BCC % (95% CI)
**Medial glenoid**	**≥ 1**	79 % (63-90)	70 % (48-85)	82 % (66-91)	67 % (45-83)
	**≥ 2**	56 % (33-77)	95 % (83-99)	82 % (52-95)	84 % (70-92)
**Lateral glenoid**	**≥ 1**	82 % (52-95)	67 % (53-80)	39 % (22-59)	94 % (79-98)
	**≥ 2**	NR (n=1)	96 % (87-99)	NR (n=1)	100 % (93-100)
**Sagittal groove**	**≥ 1**	92 % (74-98)	83 % (66-93)	81 % (63-92)	93 % (77-98)
	**≥ 2**	86 % (49-97)	89 % (77-95)	55 % (28-79)	98 % (88-100)

Table S4. Inter-modality concordance between the radiographic consensus and the MRI categorisation for subchondral bone sclerosis, by anatomical site and severity threshold. PPA = positive percent agreement; NPA = negative percent agreement; FCC = forward conditional concordance; BCC = backward conditional concordance. Cells are colour-coded by concordance level using a five-tier scale

**Poor / slight**	**Fair**	**Moderate**	**Substantial**	**Almost perfect**
< 0.20	*0.20 - 0.40*	*0.40 - 0.60*	*0.60 - 0.80*	*≥ 0.80*
