# Supplementary material for: Inter-Reader and Inter-Modality Agreement for Radiographic and Magnetic Resonance Imaging Subchondral Bone Assessment in the Equine Proximal Phalanx Is Location- and Pattern-Dependent
Source: Animals (Basel). 2026 Nov 4;16(21):3493. doi: 10.3390/ani16213493 (PMC13762259; doi:10.3390/ani16213493)
Supplement: Supplementary file 1 [file animals-16-03493-s001.zip › Table_S5.pdf]

Table S5. Source data for Figure S2 (Discordant transition profile for SCB resorption). For each reader × anatomical site, the number of fetlocks in each specific MRI category → Reader category transition is listed. MRI resorption categories: 0 = normal, 1 = poorly-defined, 2 = small defect, 3 = cyst-like, 4 = fracture. N = 54 fetlocks.

Reader	Category	Site	Discordant transitions (MRI→Reader: count)
R3	Cat1 (ECVDI)	Medial	0→1: 2; 1→0: 4; 4→1: 2
R3	Cat1 (ECVDI)	Lateral	4→1: 1
R3	Cat1 (ECVDI)	Sagittal	0→1: 2; 0→2: 1; 1→0: 2; 2→0: 2; 4→1: 1; 4→3: 1
R4	Cat1 (ECVDI)	Medial	0→1: 3; 1→0: 4; 4→0: 2; 4→1: 1
R4	Cat1 (ECVDI)	Lateral	4→1: 1
R4	Cat1 (ECVDI)	Sagittal	0→1: 2; 0→2: 1; 1→0: 2; 2→0: 2; 2→4: 5; 4→3: 1
R1	Cat2 (ECVSMR)	Medial	0→1: 2; 1→0: 3; 4→0: 1; 4→1: 2
R1	Cat2 (ECVSMR)	Lateral	4→2: 1
R1	Cat2 (ECVSMR)	Sagittal	0→1: 1; 0→2: 2; 1→0: 2; 2→0: 2; 4→1: 1; 4→2: 1; 4→3: 4
R2	Cat2 (ECVSMR)	Medial	0→1: 1; 1→0: 5; 4→0: 2; 4→2: 1
R2	Cat2 (ECVSMR)	Lateral	4→0: 1
R2	Cat2 (ECVSMR)	Sagittal	0→1: 1; 1→0: 2; 2→0: 2; 2→1: 1; 2→4: 1; 4→0: 1; 4→1: 1; 4→2: 1; 4→3: 3
R5	Cat3 (GP)	Medial	0→1: 3; 0→2: 1; 1→0: 4; 4→0: 2; 4→1: 1
R5	Cat3 (GP)	Lateral	0→2: 2; 4→0: 1
R5	Cat3 (GP)	Sagittal	0→1: 3; 1→0: 2; 2→0: 1; 2→1: 1; 2→3: 1; 2→4: 1; 4→0: 1; 4→2: 1; 4→3: 5
R6	Cat3 (GP)	Medial	0→1: 4; 1→0: 4; 4→0: 2; 4→2: 1
R6	Cat3 (GP)	Lateral	0→1: 3; 0→2: 1; 4→0: 1
R6	Cat3 (GP)	Sagittal	0→1: 5; 0→2: 3; 0→3: 2; 1→0: 2; 2→0: 1; 2→1: 1; 2→3: 1; 2→4: 1; 4→1: 1; 4→2: 1; 4→3: 3
R7	Cat3 (GP)	Medial	0→2: 2; 1→0: 6; 4→0: 3
R7	Cat3 (GP)	Lateral	4→0: 1
R7	Cat3 (GP)	Sagittal	0→1: 1; 0→2: 1; 1→0: 2; 2→0: 2; 2→1: 2; 4→0: 1; 4→2: 4; 4→3: 1
