# Supplementary material for: Inter-Reader and Inter-Modality Agreement for Radiographic and Magnetic Resonance Imaging Subchondral Bone Assessment in the Equine Proximal Phalanx Is Location- and Pattern-Dependent
Source: Animals (Basel). 2026 Nov 4;16(21):3493. doi: 10.3390/ani16213493 (PMC13762259; doi:10.3390/ani16213493)
Supplement: Supplementary file 1 [file animals-16-03493-s001.zip › Table_S6.pdf]

Site	PPA % (95 % CI)	NPA % (95 % CI)	FCC % (95 % CI)	BCC % (95 % CI)
**Medial glenoid**	56 % (27-81)	96 % (85-99)	71 % (36-92)	91 % (80-97)
**Lateral glenoid**	NR (n=1)	100 % (93-100)	NR (n=1)	100 % (93-100)
**Sagittal groove**	76 % (53-90)	89 % (75-96)	76 % (53-90)	89 % (75-96)

Table S6. Inter-modality concordance between the radiographic consensus and the MRI categorisation for any SCB resorption (≥ 1) at each anatomical site. PPA = positive percent agreement; NPA = negative percent agreement; FCC = forward conditional concordance; BCC = backward conditional concordance. Cells are colour-coded by concordance level using a five-tier scale

Poor / slight	Fair	Moderate	Substantial	Almost perfect
< 0.20	*0.20 - 0.40*	*0.40 - 0.60*	*0.60 - 0.80*	*≥ 0.80*
