# Supplementary material for: Inter-Reader and Inter-Modality Agreement for Radiographic and Magnetic Resonance Imaging Subchondral Bone Assessment in the Equine Proximal Phalanx Is Location- and Pattern-Dependent
Source: Animals (Basel). 2026 Nov 4;16(21):3493. doi: 10.3390/ani16213493 (PMC13762259; doi:10.3390/ani16213493)
Supplement: Supplementary file 1 [file animals-16-03493-s001.zip › Table_S7.pdf]

Site	Pattern	PPA % (95 % CI)	NPA % (95 % CI)	FCC % (95 % CI)	BCC % (95 % CI)
**Medial glenoid**	Normal	96 % (85-99)	56 % (27-81)	91 % (80-97)	71 % (36-92)
	Diffuse (type 1)	33 % (10-70)	90 % (78-95)	29 % (8-64)	91 % (80-97)
	Fracture (type 4)	0 % (0-56)	100 % (93-100)	NR (n=0)	94 % (85-98)
**Lateral glenoid**	Normal	100 % (93-100)	100 % (21-100)	100 % (93-100)	100 % (21-100)
	Diffuse (type 1)	NR (n=0)	98 % (90-100)	0 % (0-79)	100 % (93-100)
	Fracture (type 4)	0 % (0-79)	100 % (93-100)	NR (n=0)	98 % (90-100)
**Sagittal groove**	Normal	89 % (75-96)	76 % (53-90)	89 % (75-96)	76 % (53-90)
	Diffuse (type 1)	0 % (0-66)	96 % (87-99)	0 % (0-66)	96 % (87-99)
	Defect (type 2)	29 % (8-64)	96 % (86-99)	50 % (15-85)	90 % (79-96)
	Cyst-like (type 3)	NR (n=0)	98 % (90-100)	0 % (0-79)	100 % (93-100)
	Fracture (type 4)	88 % (53-98)	93 % (82-98)	70 % (40-89)	98 % (88-100)

Table S7. Per-pattern concordance of the radiographic consensus with the MRI categorisation for subchondral bone resorption, by anatomical site and lesion pattern. For each pattern, concordant classification was defined as the radiographic consensus assigning the same type as the MRI. PPA = positive percent agreement; NPA = negative percent agreement; FCC = forward conditional concordance; BCC = backward conditional concordance. Cells are colour-coded by concordance level using a five-tier scale

**Poor / slight**	**Fair**	**Moderate**	**Substantial**	**Almost perfect**
< 0.20	*0.20 - 0.40*	*0.40 - 0.60*	*0.60 - 0.80*	*≥ 0.80*
